# Clinicopathological Analysis of 34 Cases of Primary Antineutrophil Cytoplasmic Antibody-Associated Vasculitis in Chinese Children

**DOI:** 10.3389/fped.2021.656307

**Published:** 2021-04-26

**Authors:** Jingyi Wu, Yuxin Pei, Liping Rong, Hongjie Zhuang, Shuhan Zeng, Lizhi Chen, Xiaoyun Jiang

**Affiliations:** Department of Pediatric Nephrology and Rheumatology, The First Affiliated Hospital, Sun Yat-sen University, Guangzhou, China

**Keywords:** antineutrophil cytoplasmic antibody-associated vasculitis, glomerulonephritis, pediatric patient, clinical research, risk factors

## Abstract

**Background:** This study aimed to summarize the clinicopathological features and prognostic risk factors of primary antineutrophil cytoplasmic antibody (ANCA)-associated vasculitis (AAV) in children.

**Methods:** Clinical and prognostic data for children admitted to our center with AAV between September 2003 and September 2020 were studied retrospectively. The incidence and risk factors of end-stage renal disease (ESRD) were calculated and analyzed.

**Results:** Thirty-four children were enrolled; 28 were female, with a median onset age of 10 years. Except for one case negative for ANCA, the other 33 patients were diagnosed with microscopic polyangiitis (MPA). The most frequently involved organ was the kidney (100.0%), followed by the lungs (58.8%) and heart (50.0%). Twenty children (58.8%) progressed to ESRD with a median course of 3 months, and they were more likely to present respiratory and cardiovascular system involvement than were the non-ESRD group (*P* < 0.05). Patients in the ESRD group also had a higher serum creatinine level, 24-h protein excretion, Pediatric Vasculitis Activity Score (PVAS), and a lower level of estimated glomerular filtration rate (eGFR), hemoglobin, and complement C3 than had those in the non-ESRD group (*P* < 0.05). The main pathological manifestations were crescentic and sclerotic classes in the ESRD group and focal class in the non-ESRD group. After 6 months of induction therapy, 90.0% of cases achieved complete or partial remission. The multivariate logistic regression model showed that baseline eGFR < 60 ml/min/1.73 m^2^ was an independent risk factor for progressing to ESRD (*OR* = 0.016, 95% CI = 0.001~0.412, *P* = 0.012).

**Conclusions:** AAV in children usually occurs in teenage girls, and the most commonly involved organ is the kidney, of which hematuria is the most common symptom, followed by proteinuria, abnormal renal function (eGFR < 90 ml/min/1.73 m^2^), etc. The primary type of AAV is MPA. Nearly 60% of patients progressed to ESRD with a median course of 3 months. Baseline eGFR < 60 ml/min/1.73 m^2^ is an independent risk factor for ESRD progression in AAV children.

## Introduction

Drs. J. Charles Jennette and Ronald J. Falk first characterized the association of antineutrophil cytoplasmic antibodies (ANCAs) with necrotizing vasculitis in the 1980s and 1990s (1). ANCA-associated vasculitis (AAV) is a disease characterized by vascular inflammation, endothelial injury, and tissue damage. The main pathological changes are divided into three types: granulomatosis with polyangiitis (GPA, previously Wegener's granulomatosis), microscopic polyangiitis (MPA), and eosinophilic GPA (EGPA, previously Churg–Strauss syndrome). Myeloperoxidase-ANCA (MPO-ANCA) and proteinase 3-ANCA (PR3-ANCA) are two main autoantigen targets (2). GPA is more prevalent in European countries/regions, which is rarely observed in East Asian countries/regions. In contrast, MPA is dominant in Asian countries/regions such as China and Japan (2). The incidence of AAV is low, which is historically estimated to be 48~184 per million people, and the disease is considered as an orphan disease. However, it is not uncommon to rheumatologists, nephrologists, clinical immunologists, and other physicians (2). According to the reported data, the peak age at onset is between the fifth and seventh decade of life (3). The incidence of AAV in children, compared with adults, is even lower. Therefore, scarce information is available for the epidemiology of children with AAV (4). Thus, most of the clinical information and treatment strategies are inferred from adult evidences and applied to pediatric patients. AAV can affect multiple systems and organs. Kidney involvement is one of the most common clinical manifestations of AAV, and 20–50% of children eventually enter end-stage renal disease (ESRD) (5). In addition, respiratory and cardiovascular system involvements are common in children, which leads to a poor prognosis. There are few studies on AAV in children. Hence, it is necessary to conduct further research on this population.

This article retrospectively analyzed the clinicopathological data of 34 children with AAV in the Department of Pediatric Nephrology and Rheumatology, the First Affiliated Hospital of Sun Yat-sen University from September 2003 to September 2020, as well as the clinicopathological data and related factors affecting the prognosis, in order to provide a theoretical basis for early prevention and treatment of AAV.

## Materials and Methods

### Patients

Children with newly diagnosed AAV in the Department of Pediatric Nephrology and Rheumatology, the First Affiliated Hospital of Sun Yatsen University, from September 2003 to September 2020 were recruited consecutively into the study.

Inclusion criteria were children (1) ≤ 14 years old and (2) who fulfill the 2012 Chapel Hill Consensus Conference nomenclature for AAV (6).

Exclusion criteria were (1) secondary vasculitis; (2) tumor; (3) AAV induced by drugs such as propylthiouracil and methimazole; and (4) long-term exposure to asbestos, hydrocarbons, silica, and other environments before the onset of AAV.

Patients were divided into the ESRD group (*n* = 20) and non-ESRD (*n* = 14) group according to the eGFR. Children with eGFR lower than 15 ml/min/1.73 m^2^ or those who required maintenance dialysis or kidney transplantation during follow-up were categorized as the ESRD group. The disease activity of vasculitis in all patients was evaluated by the Pediatric Vasculitis Activity Score (PVAS) (7). There are a total of 10 items in the PVAS, including general conditions; the skin; the mucous membranes/eyes; the ears, nose and throat; the chest; the cardiovascular system; the abdomen; the kidneys; the nervous system; the others. The score is 0~63, depending on the existence or absence of clinical items for active vasculitis after excluding other causes (such as infection). The higher the score, the stronger the disease activity, with >5 points indicating vasculitis activity and ≥25 points indicating a high activity.

The prognosis referred to the criteria of the European League Against Rheumatism (EULAR) (8): complete remission (CR) was defined as renal function improved or stable, no active involvement in disease; stable extra-renal signs; no systemic inflammation; and eGFR returned to normal or slightly elevated (not directly related to the disease). Partial remission (PR) was defined as renal function, and urine test indicators were stable, extra-renal signs improved or becoming stable, and there was no progressive deterioration. Non-remission (NR) was classified as one discrepancy in the indexes mentioned above.

### Laboratory Findings

The clinical manifestation, laboratory characteristics (including leukocyte, hemoglobin, platelets, serum albumin, creatinine, eGFR, uric acid, fasting glucose, cholesterol, triglyceride, C-reactive protein, complement C3, complement C4, CD19^+^ cells, 24-h protein excretion, urinary red blood cells, positivity of antinuclear antibody, positivity of anti-double-stranded DNA antibody, and pathological characteristics at onset), treatment, and prognosis were analyzed. The normal range of CRP is 0–3 mg/L. The modified Schwartz formula was used to calculate the eGFR for each patient (9). According to the ANCA-associated glomerulonephritis (AAGN) classification system proposed by Berden et al. (10), all biopsies were classified as focal, crescentic, mixed, or sclerotic. The categories labeled focal, crescentic, and sclerotic were based on the predominance of normal glomeruli, cellular crescents, and globally sclerotic glomeruli. The mixed category represented a heterogeneous glomerular phenotype where no glomerular feature predominates. The biopsies in the focal category contained ≥50% normal glomeruli that were not affected by the disease process; the crescentic category contained biopsies with ≥50% of glomeruli with cellular crescents; the sclerotic category contained ≥50% of glomeruli with global sclerosis.

### Definitions

AAV classification was adopted from the 2017 EULAR/ACR interim classification standard (11). Serum ANCA was detected by indirect immunofluorescence (IIF) with neutrophils as a substrate combined with ELISA to detect specific target antigens (12). *Hypertension in children* (13): Blood pressure greater than the 95th percentile of the same sex, age, and height measured at least three times at different time points. *Acute kidney injury* (AKI) (14): serum creatinine (Scr) increased by both ≥20 μmol/L and ≥30% of the baseline value within 7 days. *Chronic kidney disease* (CKD) (15): renal structural or functional abnormalities or eGFR < 60 ml/min/1.73 m^2^ with a course of more than 3 months; eGFR ≥ 90 ml/min/1.73 m^2^ was CKD stage 1, 60~89 ml/min/1.73 m^2^ was CKD stage 2, 30~59 ml/min/1.73 m^2^ was CKD stage 3, 15~29 ml/min/1.73 m^2^ was CKD stage 4, and < 15 ml/min/1.73 m^2^ was CKD stage 5 or ESRD.

### Treatment Protocol

The treatment protocol was adopted from the recommendations for AAV diagnosis and treatment published by the EULAR and the European Renal Association—European Dialysis and Transplant Association (ERA-EDTA) (16), clinical practice guidelines published by Kidney Disease Improving Global Outcomes (KDIGO) (17), and review on current management for AAV children (18). Induction therapy included corticosteroids in combination with cyclophosphamide (CYC). Oral prednisone (P) was prescribed at an initial dosage of 1~2 mg/kg/day for 4~8 weeks, tapered gradually to an alternate-day dose of 0.5 mg/kg by 6 months, and then reducing doses over time until CR. CYC was administered intravenously with a dose of 8~12 mg/kg/day, consecutive for 2 days, once every 2 weeks, or 0.5~1.0 g/m^2^ once a month. Intravenous CYC was continued for 6 months. Patients with acute renal failure or pulmonary hemorrhage received three to six pulses of intravenous pulse methylprednisolone (MP) [15~30 mg/kg (maximum dose 1 g)] at initial induction therapy. Patients with severe pulmonary hemorrhage or acute renal failure or resistance to both MP/P and CYC in combination for induction therapy requiring dialysis at diagnosis underwent additional plasma exchanges (PEs) for three to six sessions. Rituximab (RTX) could also be used for induction treatment (375 mg/m^2^/week, i.v.). Low-dose prednisone (0.5 mg/kg every other day, oral) combined with immunosuppressants was used for maintenance therapy. Daily oral azathioprine (AZA) was given (0.5~2.5 mg/kg/day) for at least 2 years. The use of immunosuppressants included CYC (8~12 mg/kg/day, i.v.) and mycophenolate mofetil (MMF) (20~30 mg/kg/day, maximum dose 2 g/day, q12h, oral).

### Statistical Analysis

Normally distributed measurement data were expressed as mean ± standard deviation (SD), evaluated by the Student *t*-test. Non-normal distribution was expressed as median [interquartile range (IQR)], evaluated by the Mann–Whitney *U* test or Wilcoxon rank-sum test. Categorical variables were expressed as percentages and were tested using the Pearson chi-square test or Fisher's exact test. Logistic regression was used to analyze the risk factors of children progressing to ESRD. Results were considered significant at *P* < 0.05. Data analysis was performed with SPSS version 25.0 (SPSS, Chicago, IL, USA).

### Ethics Statement

This research was in compliance with the Declaration of Helsinki and approved by the ethics committee of the First Affiliated Hospital of Sun Yat-sen University [No. (2021)019]. Written informed consent was obtained from all of the patients' parents or guardians.

## Results

### Clinical Manifestations

Thirty-four children were diagnosed with AAV in our center from 2003 to 2019, with an average of two cases per year. The number of confirmed cases was the least from 2004 to 2008 (both one case) and the most in 2016 and 2019 (both four cases), and there was an upward trend over 17 years. Among the 34 children, six were male (17.6%) and 28 were female (82.4%). The male-to-female ratio was 1:4.7, and the average age at disease onset was 9.5 ± 0.5 years. The time from diagnosis of AAV to the last follow-up was 0.4–117.4 months, with a median time of 9.2 months. The most frequently involved organ was the kidney (100.0%), of which hematuria (94.1%) was the most common symptom, followed by proteinuria (88.2%), anemia (73.5%), and oliguria/anuria (44.1%). At the time of onset, the median eGFR of 34 patients was 23.1 ml/min/1.73 m^2^. Among them, eGFR was >90 ml/min/1.73 m^2^ in 12 children (35.3%), between 60 and 90 ml/min/1.73 m^2^ in one child (2.9%), between 15 and 60 ml/min/1.73 m^2^ in seven children (20.6%), and <15 ml/min/1.73 m^2^ in 14 children (41.2%). Respiratory system involvement (58.8%) often presented as exudative lesions (41.2%), pleural effusion/pleurisy (23.5%), pulmonary hemorrhage (17.7%), dyspnea (14.7%), etc. Cardiovascular system involvement was observed in 50.0% of children, with symptoms of pericardial effusion (26.5%), heart valve abnormalities (17.7%), etc. The eyes/ears/nose (41.2%), gastrointestinal system (17.6%), nervous system (14.7%), and skin (8.8%) were less affected. During follow-up, 20 children progressed to ESRD, in which the male-to-female ratio was 1:5.7, and the average age was 10.6 ± 0.5 years. The median time to ESRD was 3.0 (range 0.2–116.8) months. There was no significant difference in gender between the two groups (*P* > 0.05) (Table 1). Sixteen cases (47.1%) received hemodialysis therapy (HD) at the beginning of the disease (**Table 5**). The average systolic and diastolic blood pressure, the proportions of hypertension, respiratory system involvement, and cardiovascular system involvement in the ESRD group were higher than those in the non-ESRD group (*P* < 0.05) (Table 1).

**Table 1:** Comparison of clinical data between the end-stage renal disease (ESRD) group and non-ESRD group at first admission.

**Variables: n (%) or median (IQR) or mean ± SD**	**ESRD group (*n* = 20)**	**Non-ESRD group (*n* = 14)**	**Total (*n* = 34)**	***P*-value**
Age (years)	10.58 ± 0.53	7.84 ± 0.79	9.45 ± 0.50	0.006
Gender (male/female)	3/17	3/11	6/28	0.628
Clinical characteristics				
BMI (k g/m^2^)	15.82 (13.89,17.70)	14.82 (14.08,16.18)	15.64 ± 0.41	0.382
SBP (mmHg)	134.30 ± 3.20	104.71 ± 2.63	122.12 ± 3.32	<0.001
DBP (mmHg)	89.55 ± 4.18	67.71 ± 2.46	80.56 ± 3.22	<0.001
Hypertension	18 (90.0)	7 (50.0)	25 (73.5)	0.009
Fever (>38°C)	1 (5.0)	1 (7.1)	2 (5.9)	0.794
Weight loss > 5%	3 (15.0)	0 (0)	3 (8.8)	0.129
Arthralgia and arthritis	1 (5.0)	1 (7.1)	2 (5.9)	0.794
Renal findings				
Hematuria	19 (95.0)	13 (92.9)	32 (94.1)	0.794
Proteinuria	19 (95.0)	11 (78.6)	30 (88.2)	0.143
Anemia	17 (85.0)	8 (57.1)	25 (73.5)	0.070
Oliguria/anuria	12 (60.0)	3 (21.4)	15 (44.1)	0.076
AKI	5 (25.0)	4 (28.6)	9 (26.5)	0.816
Organ involvement				
Respiratory system	15 (75.0)	5 (35.7)	20 (58.8)	0.022
Cardiovascular system	15 (75.0)	2 (14.3)	17 (50.0)	<0.001
Eyes/ears/nose	7 (35.0)	7 (50.0)	14 (41.2)	0.382
Gastrointestinal system	5 (25.0)	1 (7.1)	6 (17.6)	0.179
Nervous system	4 (20.0)	1 (7.1)	5 (14.7)	0.298
Skin	1 (5.0)	2 (14.3)	3 (8.8)	0.347
PVAS	18.00 (16.50, 22.00)	13.00 (9.75, 17.25)	18.00 (12.75, 20.00)	0.004

*ESRD, end-stage renal disease; BMI, body mass index; SBP, systolic blood pressure; DBP, diastolic blood pressure; AKI, acute kidney injury; PVAS, pediatric vasculitis activity score; IQR, interquartile range; SD, standard deviation*.

### Laboratory Data

Of the 34 children, 25 were positive for P-ANCA and MPO-ANCA (73.5%), four were positive for P-ANCA (11.8%), three were positive for MPO-ANCA (8.8%), one was positive for P-ANCA and MPO-ANCA combined with PR3-ANCA (2.9%), and one was negative for ANCA (2.9%). The values of Scr, blood uric acid, fasting glucose, 24-h protein excretion, and PVAS in the ESRD group were higher than those in the non-ESRD group, while the values of eGFR, hemoglobin, platelet, serum albumin, and C3 in the ESRD group were lower than those in the non-ESRD group (*P* < 0.05) (Table 2).

**Table 2:** Comparison of laboratory examination indexes between the end-stage renal disease (ESRD) group and non-ESRD group at first admission.

**Variables: n (%) or median (IQR) or mean ± SD**	**ESRD group (*n* = 20)**	**Non-ESRD group (*n* = 14)**	***P*-value**
Leukocyte ( × 10^9^/L)	8.58 (7.50, 11.60)	7.16 (6.50, 15.11)	0.624
Hemoglobin (g/L)	73.65 ± 4.36	111.86 ± 3.66	<0.001
Platelets ( × 10^9^/L)	239.85 ± 35.63	395.14 ± 38.54	0.007
Serum albumin (g/L)	30.27 ± 1.32	37.08 ± 1.83	0.004
Creatinine (μmol/L)	598.50 (431.00, 872.25)	42.50 (32.00, 59.00)	<0.001
eGFR (ml/min/1.73 m^2^)	11.15 (8.18, 15.93)	141.64 (116.57, 177.27)	<0.001
Uric acid (μmol/L)	433.00 (351.50, 587.50)	247.00 (200.00, 392.50)	0.002
Fasting glucose (mmol/L)	4.80 (4.18, 5.70)	4.20 (3.85, 4.75)	0.042
Cholesterol (mmol/L)	5.30 (4.53, 7.30)	5.00 (4.25, 6.68)	0.539
Triglyceride (mmol/L)	2.01 (1.23, 3.13)	1.17 (0.97, 2.08)	0.060
C-reactive protein (mg/L)	3.25 (1.00, 7.77)	3.83 (1.00, 8.39)	0.836
Complement C3 (g/L)	0.86 (0.72, 0.91)	1.15 (0.91, 1.31)	0.018
Complement C4 (g/L)	0.24 ± 0.02	0.24 ± 0.02	0.909
CD19^+^ cells (%)	26.33 ± 3.38	20.81 ± 1.71	0.226
24-h protein excretion (g)	1.71 (1.08, 2.24)	0.51 (0.23, 1.20)	0.030
urinary red blood cells (/μl)	191.40 (27.28, 464.73)	222.00 (75.00, 405.90)	0.787
Positive antinuclear antibody	10 (55.6)	5 (35.7)	0.265
Positive anti-double-stranded DNA antibody	6 (33.3)	3 (21.4)	0.457

*ESRD, end-stage renal disease; eGFR, estimated glomerular filtration rate; CD19^+^ cells, CD19^+^ B lymphocytes in peripheral blood; IQR, interquartile range; SD, standard deviation*.

### Renal Histopathology

Renal biopsy was performed in 30 children, including 17 cases in the ESRD group and 13 cases in the non-ESRD group. The remaining four patients' parents refused the renal biopsy. According to the Berden classification, there were 13 cases of focal (43.3%), eight cases of crescentic (26.7%), two cases of mixed (6.7%), and seven cases of sclerotic classes (23.3%). The patients' biopsies in the ESRD group were mainly crescentic and sclerotic, while those in the non-ESRD group were mainly focal (*P* = 0.001). The proportion of crescentic, mixed, and sclerotic classes in the ESRD group was higher than that in the non-ESRD group, while the proportion of focal class was lower. The proportion of glomerular sclerosis was higher and the proportion of normal glomerulus, mesangial cells, and matrix hyperplasia was lower in the ESRD group compared with that in the non-ESRD group (*P* < 0.05). The deposition of immunofluorescence included IgM, IgG, IgA, C1q, and C3, of which C3 deposition was dominant. There was no significant difference between the two groups (*P* > 0.05) (Table 3).

**Table 3:** Comparison of renal pathology between the end-stage renal disease (ESRD) group and non-ESRD group.

**Variables: n (%) or median (IQR) or mean ± SD**	**ESRD group (*n* = 17)**	**Non-ESRD group (*n* = 13)**	**Total (*n* = 30)**	***P-*value**
**Berden classification**				
Focal	2 (11.8)	11 (84.6)	13 (43.3)	0.001
Crescentic	6 (35.3)	2 (15.4)	8 (26.7)	
Mixed	2 (11.8)	0 (0)	2 (6.7)	
Sclerotic	7 (41.2)	0 (0)	7 (23.3)	
**Renal biopsy findings**				
Normal glomerulus (%)	11.1 (0, 22.2)	66.7 (47.4, 75.0)	23.0 (8.2, 66.9)	<0.001
Glomerular sclerosis (%)	53.3 (23.2, 63.4)	0 (0, 13.4)	27.4 (0, 56.7)	<0.001
Crescents (%)	30.4 ± 5.7	21.4 ± 3.5	26.5 ± 3.6	0.189
Fibrinoid necrosis	0 (0)	1 (7.7)	1 (3.3)	0.245
Segmental sclerosis	7 (41.2)	7 (53.8)	14 (46.7)	0.491
Mesangial cell and matrix hyperplasia	5 (29.4)	10 (76.9)	15 (50.0)	0.010
Immunofluorescence				
IgM	4 (23.5)	6 (46.2)	10 (33.3)	0.193
IgG	4 (23.5)	1 (7.7)	5 (16.7)	0.249
IgA	1 (5.9)	3 (23.1)	4 (13.3)	0.170
C1q	5 (29.4)	1 (7.7)	6 (20.0)	0.141
C3	10 (58.8)	5 (38.5)	15 (50.0)	0.269
Renal tubular atrophy	12 (70.6)	6 (46.2)	18 (60.0)	0.176
Interstitial fibrosis	12 (70.6)	6 (46.2)	18 (60.0)	0.176
Renal interstitial inflammatory cell infiltration	13 (76.5)	10 (76.9)	23 (76.7)	0.977

*ESRD, end-stage renal disease; IQR, interquartile range; SD, standard deviation*.

### Risk Factor Affecting the Progression of End-Stage Renal Disease

The results of the univariate logistic regression model suggested that baseline eGFR < 60 ml/min/1.73 m^2^, initial PVAS > 15, hypertension, glomerular sclerosis >50% of total glomerulus count, and mesangial cell and matrix hyperplasia were associated with the progression to ESRD in AAV children (*P* < 0.05). The further multivariate logistic regression model demonstrated that baseline eGFR < 60 ml/min/1.73 m^2^ was the independent risk factor for progression to ESRD in children with AAV (*OR* = 0.016, 95% CI = 0.001~0.412, *P* = 0.012) (Table 4).

**Table 4:** Logistic regression model with factors possibly associated with end-stage renal disease.

**Influencing factors**	**Univariate analysis**			**Multivariate analysis**		
	***OR***	**95% CI**	***P-*value**	***OR***	**95% CI**	***P-*value**
Baseline eGFR < 60 ml/min/1.73 m^2^ (yes/no)	0.009	0.001–0.114	<0.001	0.016	0.001–0.412	0.012
PVAS > 15 (yes/no)	0.071	0.013–0.382	0.002	0.783	0.033–18.533	0.880
Hypertension (yes/no)	0.111	0.018–0.671	0.017	0.193	0.009–4.190	0.295
Glomerular sclerosis >50% of total glomerular count (yes/no)	0.074	0.008–0.704	0.023	0.224	0.012–4.169	0.316
Mesangial cell and matrix hyperplasia (yes/no)	0.185	0.038–0.893	0.036	0.400	0.022–7.134	0.533

### Treatment

Children in induction therapy were treated with oral prednisone, pulse MP, and CYC. MP pulse therapy was performed in 11 patients with AKI and active pulmonary hemorrhage. Two cases were treated with RTX. Low-dose prednisone combined with immunosuppressants was used in the maintenance therapy period, including seven cases of CYC, six cases of MMF, and one case of AZA (14 children were lost to 6 months' follow-up, and seven children underwent kidney transplantation). Eleven cases (32.4%) received intravenous immunoglobulin therapy. Five cases (14.7%) were treated with plasmapheresis for three to six sessions, including two cases of pulmonary hemorrhage, one case of AKI, one case of progressive deterioration of renal function with severe systemic vasculitis and severe infection, and one case of recurrent AAGN (Table 5).

**Table 5:** Treatment of 34 patients with ANCA-associated vasculitis.

**No**.	**Gender**	**Age (years)**	**Drug therapy**		**Renal replacement therapy**		**PVAS 1**	**PVAS 2**	**Induction therapy response**	**Last follow-up outcome**
			**Induction therapy (for 6 months)**	**Maintenance therapy (after 6 months)**	**At onset**	**Last follow-up**				
1	F	3.0	P, MP, CYC	P, MP, MMF, IVIG	-	-	29	4	CR	CR
2	F	8.6	P	^*^	HD	-	18	^*^	^*^	ESRD
3	F	12.4	P, MP, CYC, IVIG	-	HD	RT	20	3	PR	ESRD
4	F	3.6	P, MP, CYC	P, MP, CYC, AZA	-	HD	18	7	NR	ESRD
5	M	9.2	P, MP, CYC	^*^	PD	-	22	D	D	D
6	F	10.9	P	-	HD	RT	18	4	PR	ESRD
7	F	3.0	^#^	^*^	-	-	12	^*^	^*^	PR
8	F	8.3	P, MP, CYC	-	HD	RT	18	3	PR	ESRD
9	F	11.0	P, MP, CYC	-	HD	RT	22	2	PR	ESRD
10	F	13.3	IVIG	^*^	HD	-	22	^*^	^*^	ESRD
11	F	5.0	P, MP, CYC, MMF	P, MMF	-	-	14	7	NR	D
12	F	11.4	P, MP, CYC, PE	-	HD	RT	14	6	PR	ESRD
13	F	8.0	P, MP, CYC, IVIG	^*^	HD	-	19	D	D	D
14	F	14.1	P, MP, CYC, IVIG, PE	^*^	HD	-	16	^*^	^*^	ESRD
15	F	11.7	P, MP, CYC	^*^	HD	-	12	^*^	^*^	ESRD
16	F	10.1	P, CYC	-	HD	RT	18	4	PR	ESRD
17	M	7.0	P, MP, CYC, MMF	P, MMF	-	-	11	5	PR	PR
18	F	13.0	P, CYC	^*^	HD	-	16	^*^	^*^	ESRD
19	F	7.6	P, MP, CYC, IVIG	P, MMF	-	-	12	3	PR	PR
20	F	9.3	P, MP, CYC	P, MMF	-	-	14	0	CR	PR
21	M	13.4	P, CYC, PE	P, CYC	HD	HD	22	10	PR	ESRD
22	F	10.0	IVIG	^*^	-	-	18	D	D	D
23	M	11.6	P, MP, CYC, IVIG, PE	-	HD	RT	22	10	PR	ESRD
24	F	9.9	P, MP, CYC, IVIG, PE	^*^	HD	-	22	^*^	^*^	ESRD
25	F	4.4	P, MP, CYC, RTX, IVIG	^*^	-	-	18	^*^	^*^	PR
26	F	10.4	P, MP, CYC, MMF, IVIG	P, MMF	-	-	20	4	PR	CKD3
27	M	10.0	P, MP, CYC	P, MP, CYC	-	-	6	2	PR	PR
28	F	10.6	P, MP, CYC	P, CYC	-	-	1	0	CR	CR
29	F	11.8	P, MP, CYC	P, CYC	-	-	6	0	CR	CR
30	F	11.0	P, MP, CYC	^*^	-	-	19	^*^	^*^	ESRD
31	F	8.2	P, MP, CYC	P, CYC	-	-	13	0	CR	CR
32	M	10.8	P, MP, CYC	P, CYC	-	-	13	0	CR	CR
33	F	10.0	-	^*^	HD	-	12	^*^	^*^	ESRD
34	F	8.7	P, CYC, RTX	^*^	-	-	17	^*^	^*^	PR

*PVAS, pediatric vasculitis activity score; PVAS1, PVAS at onset; PVAS2, PVAS at 6 months; P, oral prednisone; MP, pulse methylprednisolone; CYC, cyclophosphamide; HD, hemodialysis; PD, peritoneal dialysis; MMF, mycophenolate mofetil; RTX, rituximab; AZA, azathioprine; IVIG, intravenous immunoglobulin; PE, plasmapheresis; RT, renal transplantation; CR, complete remission; PR, partial remission; NR, non-remission; D, death; CKD3, chronic kidney disease stage 3; ESRD, end-stage renal disease; ANCA, antineutrophil cytoplasmic antibody*.

# *Never used steroids, only anticoagulation, anti-inflammatory, etc*.

* *Course of treatment did not exceed 6 months*.

### Prognosis

At the time of onset, 22 children had eGFR < 90 ml/min/1.73 m^2^. There were 10 and 20 children who progressed to ESRD at 6 months and the end of follow-up, respectively. During the first 6 months of follow-up, three children died and 11 children were lost to follow-up. After 6 months of treatment, six cases had CR, 12 cases had PR, and two cases had NR. Among those with PR, three cases were diagnosed ESRD after disease onset and achieved extra-renal remission followed by kidney transplantation, and another four cases underwent kidney transplantation due to ESRD during follow-up. The median PVAS was 19 at the onset and 3 at 6 months after treatment. The median PVAS of 12 cases of follow-up outcome was 1, with seven cases of renal transplantation and one dead child, indicating that the PVAS decreased significantly after treatment. At the end of follow-up, four cases died. Among them, three (8.8%) deaths were in the ESRD group, which resulted from hypertensive encephalopathy (one case), cerebrovascular accident (one case), and uremia due to cessation of treatment (one case). The only one death (2.9%) in the non-ESRD group was due to intracranial hemorrhage and pulmonary hemorrhage (Table 5).

## Discussion

AAV occurs mainly in adults and is a rare disease in the pediatric population. The incidence of AAV in adults in Europe is 13~20/1 million (4). In some pediatric rheumatic clinics in the United States, 3% of children were diagnosed with primary pediatric vasculitis (19). However, the incidence of AAV in children is on the rise in recent years. Girls are mostly affected, with an overall average age of 10.7–14.0 years at the time of diagnosis (20). And MPA is the leading cause of AAV in children (21). In this study, a total of 28 female children (82.4%) were diagnosed with AAV at between 3.0 and 14.1 years old, with an average of (9.5 ± 0.5) years old, and the incidence increased year by year, suggesting that adolescent girls were getting more commonly affected. In this study, 97.1% of the children had MPA, which was consistent with previous studies (21).

Patients with AAV often present with multiple organ abnormalities, including in the kidneys, lungs, skin, and joints (22). The most frequently affected organ by MPA is the kidney. All children in this study had kidney involvement, who manifested with hematuria, proteinuria, anemia, and AKI. Lung and heart involvements were the second and third most common and severe. The proportion of lung and heart involvements in the ESRD group was much higher than that in the non-ESRD group, with severer clinical symptoms, indicating that the degree of cardiopulmonary damage in the ESRD group was greater than that in the non-ESRD group; thus, the prognosis was worse. The values of Scr and 24-h urine protein quantification in the ESRD group were higher, while the serum albumin and eGFR values were lower than in the non-ESRD group, indicating that the ESRD group had severer kidney involvement at disease onset. These may be risk factors for poor renal prognosis. The level of complement C3 in the ESRD group was also lower than that in the non-ESRD group (*P* = 0.018). There was no significant difference in the level of complement C4 between the two (*P* > 0.05) (Table 2). The results were consistent with the study of Augusto et al. (23). It had been reported that C3 level might be a potential biomarker reflecting the prognosis of the kidney, while complement C4 might not be related to the prognosis of AAV. Additionally, C3 deposition could be seen in the glomerulus of children with AAV, indicating that complement pathway abnormalities may be involved in the pathogenesis of AAV (24, 25).

The PVAS was released in 2012, redefining the Birmingham Vasculitis Activity Score (BVAS). This score is a useful tool in pediatric patients with systemic vasculitis and can be used in clinical trials to determine disease activity (7). In this study, the PVAS of the ESRD group was higher than that of the non-ESRD group [18.0 (16.5, 22.0) vs. 13.0 (9.8, 17.3)], suggesting that the activity of vasculitis in the ESRD group was significantly higher. Patients with high PVAS are more likely to progress to ESRD. Previous studies have shown that BVAS could be used as a risk factor for poor prognosis of adult AAV patients and could predict relapse during the follow-up in patients with GPA and MPA (26–32). In this study, the univariate logistic regression model showed that initial PVAS > 15 was a risk factor for progression to ESRD in children with AAV (*P* < 0.05), which was in line with previous research results (26–32). In addition, the PVAS at onset was higher than the PVAS at 6 months [18.0 (12.8, 20.0) vs. 3.5 (0.5, 5.8)] (*P* < 0.05) (Table 5), suggesting that the PVAS might have the ability to reflect disease activity and therapeutic effect of AAV in children.

Some studies have proved that the prognosis of focal class is the best according to the Berden classification, while the prognosis of sclerotic class is the worst (33). In this study, crescentic and sclerotic classes were dominant in the ESRD group, while focal class was dominant in the non-ESRD group. The prognosis of focal class was relatively good, but that of crescentic and sclerotic was poor, indicating that Berden classification could predict the prognosis of AAV. Therefore, early detection and timely renal puncture are essential in guiding AAV treatment, protecting renal function and even reversing inflammatory response and preventing irreversible renal damage.

Previous studies have reported that baseline eGFR < 60 ml/min/1.73 m^2^, hypertension, baseline Scr, oliguria/anuria, histopathological classification, and renal tubular atrophy are risk factors for poor prognosis in AAV (33–41). In this study, the univariate analysis showed that baseline eGFR < 60 ml/min/1.73 m^2^, hypertension, glomerular sclerosis >50% of total glomerular count, mesangial cell, and matrix hyperplasia were risk factors for progression to ESRD in AAV children (*P* < 0.05). Multivariate analysis further determined that baseline eGFR < 60 ml/min/1.73 m^2^ was an independent risk factor for ESRD (*OR* = 0.016, 95% CI = 0.001~0.412, *P* = 0.012). These results are consistent with previous studies that patients with decreased eGFR had poor kidney prognosis (38–41). To date, there are no large pediatric studies on AAV. Therefore, we compared our results with adult/pediatric combined studies revealing that impaired kidney function at onset, including glomerular sclerosis >50% of total glomerular count, and mesangial cell and matrix hyperplasia, were risk factors for ESRD. Additionally, we also found that hypertension at baseline was a strong predictor of ESRD. These results can predict the progression of renal function to ESRD in children with AAV. However, the results of this study are not entirely consistent with previous studies (33–37), primarily because of the small sample size, which did not meet the requirements of events per variable (EPV). Although the results might not be stable enough, they were reported considering the rare incidence and absent clinical evidences in children. The reliability of the results required further verification by randomized large-scale studies.

The management strategies of AAV in children are inferred from adult experiences and studies. Steroids combined with CYC are used in induction therapy, and low-dose steroids combined with immunosuppressants are used for maintenance therapy (16, 42). With the current evidence of the pathogenesis of AAV, the application of RTX, MMF, and PE in AAV further improved the therapeutic effect of AAV. In this study, the children were treated with steroids combined with CYC during the induction period, while those with AKI and active pulmonary hemorrhage were treated with high-dose MP. Low-dose steroids, combined with immunosuppressants, such as CYC, MMF, RTX, and AZA, were used during the maintenance period. Patients with pulmonary hemorrhage, AKI, progressive deterioration of renal function, and recurrence of AAGN were treated with plasmapheresis. Some children who progressed to ESRD received renal transplantation. Treatment responses were evaluated after 6 months. There were six cases of CR, 12 cases of PR, and two cases of no remission. Children who received PE had improved renal function. Therefore, this study provides some evidence in treating AAV children according to EULAR/ERA-EDTA and KDIGO guidelines with oral prednisone combined with CYC or other immunosuppressants for induction and low-dose prednisone combined with RTX or AZA for maintenance. In addition, PE and hemodialysis can be used in severe conditions. Clinicians should thoroughly evaluate and balance the benefits and risks based on the characteristics of the disease, the degree of disease activity, the involved systems, and comorbidities before formulating the best individualized treatment.

This study also has some limitations. It is a single-center retrospective study with a small sample size and simple statistical methods. Large-scale multicenter studies and prospective cohort studies are of importance for better understanding the clinical characteristics, treatment options, and prognosis of children with AAV.

In conclusion, AAV is a rare childhood systemic vasculitis accompanied by severe organ damage. MPA is the most common type of AAV in children. Among the renal pathologic injuries, sclerotic and crescentic classes are more severe, with unfavorable prognoses. C3 level may become a potential biomarker to reflect the prognosis of the kidney. Patients with a high PVAS are more likely to progress to ESRD. eGFR < 60 ml/min/1.73 m^2^ at disease onset is an independent risk factor for ESRD.

## Data Availability Statement

The original contributions presented in the study are included in the article/supplementary material, further inquiries can be directed to the corresponding author/s.

## Ethics Statement

The studies involving human participants were reviewed and approved by the ethics committee of the First Affiliated Hospital of Sun Yat-sen University. Written informed consent to participate in this study was provided by the participants' legal guardian/next of kin.

## Author Contributions

XJ and LC designed the study and reviewed and revised the manuscript. JW and YP carried out the initial analyses and drafted the initial manuscript. LR, HZ, and SZ coordinated and supervised the data collection. All authors contributed to the article and approved the submitted version.

## Conflict of Interest

The authors declare that the research was conducted in the absence of any commercial or financial relationships that could be construed as a potential conflict of interest.

## References

[1] Jennette JC Falk RJ. Antineutrophil cytoplasmic autoantibodies and associated diseases: a review. Am. J. Kidney Dis. (1990) 15:517–29. 10.1016/S0272-6386(12)80521-X 1973331

[2] Kitching AR Anders HJ Basu N Brouwer E Gordon J Jayne DR. ANCA-associated vasculitis. Nat. Rev. Dis. Primers. (2020) 6:71. 10.1038/s41572-020-0204-y 32855422

[3] Kamesh L Harper L Savage CO. ANCA-positive vasculitis. J. Am. Soc. Nephrol. (2002) 13:1953–60. 10.1097/01.ASN.0000016442.33680.3E 12089393

[4] Jariwala MP Laxer RM. Primary vasculitis in childhood: GPA and MPA in childhood. Front. Pediatr. (2018) 6:226. 10.3389/fped.2018.00226 30167431 PMC6107029

[5] Chen Y Chen X. Antineutrophil cytoplasmic antibodies-associated glomerulonephritis: from bench to bedside. Chronic Dis. Transl. Med. (2018) 4:187–91. 10.1016/j.cdtm.2018.05.004 30276365 PMC6160504

[6] Jennette JC Falk RJ Bacon PA Basu N Cid MC Ferrario F. 2012 revised International Chapel Hill Consensus Conference nomenclature of vasculitides. Arthritis Rheum. (2013) 65:1–11. 10.1002/art.37715 23045170

[7] Dolezalova P Price-Kuehne FE Özen S Benseler SM Cabral DA Anton J. Disease activity assessment in childhood vasculitis: development and preliminary validation of the Paediatric Vasculitis Activity Score (PVAS). Ann. Rheum. Dis. (2013) 72:1628–33. 10.1136/annrheumdis-2012-202111 23100606

[8] Hellmich B Flossmann O Gross WL Bacon P Cohen-Tervaert JW Guillevin L. EULAR recommendations for conducting clinical studies and/or clinical trials in systemic vasculitis: focus on anti-neutrophil cytoplasm antibody-associated vasculitis. Ann. Rheum. Dis. (2007) 66:605–17. 10.1136/ard.2006.062711 17170053 PMC2703775

[9] Kouri AM Andreoli SP. Clinical presentation and outcome of pediatric ANCA-associated glomerulonephritis. Pediatr. Nephrol. (2017) 32:449–55. 10.1007/s00467-016-3490-6 27677979

[10] Berden AE Ferrario F Hagen EC Jayne DR Jennette JC Joh K. Histopathologic classification of ANCA-associated glomerulonephritis. J. Am. Soc. Nephrol. (2010) 21:1628–36. 10.1681/ASN.2010050477 20616173

[11] Yoo J Kim HJ Ahn SS Jung SM Song JJ Park YB. The utility of the ACR/EULAR 2017 provisional classification criteria for granulomatosis with polyangiitis in Korean patients with antineutrophil cytoplasmic antibody-associated vasculitis. Clin. Exp. Rheumatol. (2018) 36(Suppl. 111):85–7. 29185957

[12] Siomou E Tramma D Bowen C Milford DV. ANCA-associated glomerulonephritis/systemic vasculitis in childhood: clinical features-outcome. Pediatr. Nephrol. (2012) 27:1911–20. 10.1007/s00467-012-2198-5 22648163

[13] Flynn JT Kaelber DC Baker-Smith CM Blowey D Carroll AE Daniels SR. Clinical practice guideline for screening and management of high blood pressure in children and adolescents. Pediatrics. (2017) 140:e20171904. 10.1542/peds.2017-3035 28827377

[14] Xu X Nie S Zhang A Jianhua M Liu H Xia H. A new criterion for pediatric aki based on the reference change value of serum creatinine. J. Am. Soc. Nephrol. (2018) 29:2432–42. 10.1681/ASN.2018010090 30054338 PMC6115652

[15] Levin A Stevens PE. Summary of KDIGO 2012 CKD guideline: behind the scenes, need for guidance, and a framework for moving forward. Kidney Int. (2014) 85:49–61. 10.1038/ki.2013.444 24284513

[16] Yates M Watts RA Bajema IM Cid MC Crestani B Hauser T. EULAR/ERA-EDTA recommendations for the management of ANCA-associated vasculitis. Ann. Rheum. Dis. (2016) 75:1583–94. 10.1136/annrheumdis-2016-209133 27338776

[17] Cattran DC Feehally J Cook HT Liu ZH Fervenza FC Mezzano SA. Kidney disease: improving global outcomes (KDIGO) glomerulonephritis work group. KDIGO clinical practice guideline for glomerulonephritis. Kidney Int. Suppl. (2012) 2:139–274. 10.1038/kisup.2012.923871408

[18] Plumb LA Oni L Marks SD Tullus K. Paediatric anti-neutrophil cytoplasmic antibody (ANCA)-associated vasculitis: an update on renal management. Pediatr. Nephrol. (2018) 33:25–39. 10.1007/s00467-016-3559-2 28062909 PMC5700225

[19] Iudici M Puéchal X Pagnoux C Quartier P Agard C Aouba A. Brief report: childhood-onset systemic necrotizing vasculitides: long-term data from the French vasculitis study group registry. Arthritis Rheumatol. (2015) 67:1959–65. 10.1002/art.39122 25808634

[20] Novick TK Chen M Scott J Cortazar FB Ayoub I Little MA. Patient outcomes in renal-limited antineutrophil cytoplasmic antibody vasculitis with inactive histology. Kidney Int. Rep. (2018) 3:671–6. 10.1016/j.ekir.2018.01.012 29854975 PMC5976815

[21] Calatroni M Oliva E Gianfreda D Gregorini G Allinovi M Ramirez GA. ANCA-associated vasculitis in childhood: recent advances. Ital. J. Pediatr. (2017) 43:46. 10.1186/s13052-017-0364-x 28476172 PMC5420084

[22] Jariwala M Laxer RM. Childhood GPA, EGPA, and MPA. Clin. Immunol. (2020) 211:108325. 10.1016/j.clim.2019.108325 31837445

[23] Augusto JF Langs V Demiselle J Lavigne C Brilland B Duveau A. Low serum complement C3 levels at diagnosis of renal ANCA-associated vasculitis is associated with poor prognosis. Plos ONE. (2016) 11:e158871. 10.1371/journal.pone.0158871 27391243 PMC4938207

[24] Brilland B Garnier A Chevailler A Jeannin P Subra J Augusto J. Complement alternative pathway in ANCA-associated vasculitis: two decades from bench to bedside. Autoimmun. Rev. (2020) 19:102424. 10.1016/j.autrev.2019.102424 31734405

[25] Hutton HL Holdsworth SR Kitching AR. ANCA-associated vasculitis: pathogenesis, models, and preclinical testing. Semin. Nephrol. (2017) 37:418–35. 10.1016/j.semnephrol.2017.05.016 28863790

[26] Yoo J Kim HJ Ahn SS Jung SM Song JJ Park YB. Clinical and prognostic features of Korean patients with MPO-ANCA, PR3-ANCA and ANCA-negative vasculitis. Clin. Exp. Rheumatol. (2017) 35(Suppl. 103):111–8. 28339364

[27] Oh YJ Ahn SS Park ES Jung SM Song JJ Park YB. Chest and renal involvements, Birmingham vascular activity score more than 13.5 and five factor score. (1996) more than 1 at diagnosis are significant predictors of relapse of microscopic polyangiitis. Clin. Exp. Rheumatol. (2017) 35(Suppl. 103):47–54. 28134074

[28] Bai YH Li ZY Chang DY Chen M Kallenberg CG Zhao MH. The BVAS is an independent predictor of cardiovascular events and cardiovascular disease-related mortality in patients with ANCA-associated vasculitis: a study of 504 cases in a single Chinese center. Semin. Arthritis. Rheum. (2018) 47:524–9. 10.1016/j.semarthrit.2017.07.004 28917713

[29] Yoo J Ahn SS Jung SM Song JJ Park YB Lee SW. Delta neutrophil index is associated with vasculitis activity and risk of relapse in ANCA-associated vasculitis. Yonsei. Med. J. (2018) 59:397–405. 10.3349/ymj.2018.59.3.397 29611402 PMC5889992

[30] Chen Z Lin L Yang W Chen N Lin Y. Clinical characteristics and prognostic risk factors of anti-neutrophil cytoplasmic antibody (ANCA)-associated vasculitides (AAV). Int. Immunopharmacol. (2020) 87:106819. 10.1016/j.intimp.2020.106819 32717565

[31] Intapiboon P Siripaitoon B. Thai patients with antineutrophil cytoplasmic antibody-associated vasculitis: outcomes and risk factors for mortality. J. Clin. Rheumatol. (2020) 18. 10.1097/RHU.0000000000001456 32568951

[32] Zhang Z Liu S Guo L Wang L Wu Q Zheng W. Clinical characteristics of peripheral neuropathy in eosinophilic granulomatosis with polyangiitis: a retrospective single-center study in China. J. Immunol. Res. (2020) 2020:3530768. 10.1155/2020/3530768 32714994 PMC7355369

[33] Chang DY Wu LH Liu G Chen M Kallenberg CGM Zhao MH. Re-evaluation of the histopathologic classification of ANCA-associated glomerulonephritis: a study of 121 patients in a single center. Nephrol. Dial. Transpl. (2012) 27:2343–9. 10.1093/ndt/gfr643 22121235

[34] Wu T Zhong Y Zhou Y Chen J Yang Y Tang R. Clinical characteristics and prognosis in 269 patients with antineutrophil cytoplasimc antibody associated vasculitis. Zhong Nan Da Xue Xue Bao Yi Xue Ban. (2020) 45:916–22. 10.11817/j.issn.1672-7347.2020.190436 33053532

[35] Khalighi MA Wang S Henriksen KJ Bock M Keswani M Chang A. Pauci-immune glomerulonephritis in children: a clinicopathologic study of 21 patients. Pediatr. Nephrol. (2015) 30:953–9. 10.1007/s00467-014-2970-9 25669759

[36] Li X Liang S Zheng C Zeng C Zhang H Hu W. Clinicopathological characteristics and outcomes of pediatric patients with systemic small blood vessel vasculitis. Pediatr. Nephrol. (2014) 29:2365–71. 10.1007/s00467-014-2885-5 25027576

[37] Tanna A Guarino L Tam FW Rodriquez-Cubillo B Levy JB Cairns TD. Long-term outcome of anti-neutrophil cytoplasm antibody-associated glomerulonephritis: evaluation of the international histological classification and other prognostic factors. Nephrol. Dial. Transplant. (2015) 30:1185–92. 10.1093/ndt/gfu237 25016608

[38] Caliskan Y Torun ES Tiryaki TO Oruc A Ozluk Y Akgul SU. Immunosuppressive treatment in C3 glomerulopathy: is it really effective? Am. J. Nephrol. (2017) 46:96–107. 10.1159/000479012 28700996

[39] Bomback AS Santoriello D Avasare RS Regunathan-Shenk R Canetta PA Ahn W. C3 glomerulonephritis and dense deposit disease share a similar disease course in a large United States cohort of patients with C3 glomerulopathy. Kidney Int. (2018) 93:977–85. 10.1016/j.kint.2017.10.022 29310824

[40] Zoshima T Suzuki K Suzuki F Hara S Mizuguchi K Ito K. ANCA-associated nephritis without crescent formation has atypical clinicopathological features: a multicenter retrospective study. Clin. Exp. Nephrol. (2020) 24:999–1006. 10.1007/s10157-020-01925-5 32651749

[41] Pinarbaşi AS Dursun I Gokce I Çomak E Saygili S Bayram MT. Predictors of poor kidney outcome in children with C3 glomerulopathy. Pediatr. Nephrol. (2021) 36:1195–205. 10.1007/s00467-020-04799-72021 33130981

[42] McGeoch L Twilt M Famorca L Bakowsky V Barra L Benseler SM. CanVasc recommendations for the management of antineutrophil cytoplasm antibody-associated vasculitides. J. Rheumatol. (2016) 43:97–120. 10.3899/jrheum.150376 26523024

